# Suicide mortality among older adults in Brazil between 2000 and 2019 - estimates from the Global Burden of Disease Study 2019

**DOI:** 10.1590/0037-8682-0322-2021

**Published:** 2022-01-28

**Authors:** Ademar Moreira Pires, Júlia Gondim Maia Reis, Flávia Megda Garcia, Guilherme Augusto Veloso, Ana Paula Souto Melo, Mohsen Naghavi, Valéria Maria de Azeredo Passos

**Affiliations:** 1 Faculdade de Ciências Médicas de Minas Gerais, Programa de Pós-Graduação em Ciências da Saúde, Belo Horizonte, MG, Brasil.; 2 Faculdade Ciências Médicas de Minas Gerais, Faculdade de Medicina, Belo Horizonte, MG, Brasil.; 3 Universidade Federal de Minas Gerais, Instituto de Ciências Exatas, Programa de Pós-Graduação em Estatística, Belo Horizonte, MG, Brasil.; 4 Universidade Federal de São João del-Rei, Faculdade de Medicina, Divinópolis, MG, Brasil.; 5 Washington University, Institute of Health Metrics and Evaluation, Seattle, USA.

**Keywords:** Suicide, Epidemiology, Public health, Aged

## Abstract

**INTRODUCTION:**

Older adults present a higher risk of suicide, and Brazil is experiencing a fast population aging. To understand the impact of demographic transition, we compared Brazilian suicide mortality rates (MR) among adults (50+ years) with global rates, those from one high-income country, and those from one middle-income country. Looking for regional disparities, the MR was analyzed among older adults (60+ years) by Brazilian states.

**METHODS:**

This was an ecological study based on estimates from the Global Burden of Disease Study, from 2000 to 2019. Age-standardized MR and age-specific MR per 100,000 inhabitants were described, with 95% uncertainty intervals (UI).

**RESULTS:**

During the period, the annual estimates and the declining trend in mortality were higher in the world than in the studied countries. In 2019, global age-standardized MR was 9.39 (95% UI 8.48-10.29), compared to 5.68 (95% UI 5.40-6.19), 6.01 (95% UI 5.10-7.04), and 6.63 (95% UI 6.43-6.95) in Brazil, Mexico, and England, respectively. In Brazil, despite a significant decline in national rates, stability was observed in 15 states. An increase in aging was only found for men, who presented 3-4 times higher MR than women. The states’ rates presented large differences: in 2019, the rates among men aged 60-64 years varied from 7.24 (95% UI 5.31; 9.85) to 26.32 (95% UI 20.21; 34.50).

**CONCLUSIONS:**

The smaller decline in suicide mortality among older Brazilian adults, the increasing risk with aging, and the higher mortality among men indicate the need for specific prevention policies. The variation within states suggests differences in the data quality or in socio-cultural and historical aspects, which requires further investigation.

## INTRODUCTION

Older adults have the highest rates of suicide globally, with a greater risk for those aged 70 years and older^1^. Monitoring mortality is one of the main concerns of the programs of suicide prevention^1^ and allows the United Nations Member States to verify the success in reaching one of the Sustainable Development Goals, the reduction in one third of the suicide mortality rate between 2015 and 2030^2^. In Brazil, there are still no public policies of suicide prevention among the older adults, despite important initiatives, such as the National Policy for the Elderly from 2006^3^ and the National Policy for Prevention of Self-Mutilation and Suicide from 2019^4^.

Few studies have been conducted in Brazil on suicide among older adults and focus especially on the decades of 1990 and 2000^5^ ^-^ ^10^. A mixed ecological study of suicide among Brazilian adults of 60+ years from 2000 to 2014 identified a rising age-standardized mortality rate (ASMR) during the period, as well as clusters of higher rates in the southern states^10^.

Since 1950, Brazil has been experimenting a fast demographic aging process in a context of scarce resources and great social inequalities^11^. While it took a century for the proportion of older adults to increase from 7% to 14% of the total population in the high-income countries, this same demographic transition is expected to occur in Brazil between 2011 and 2031^12^. 

Improvement in the quality of the estimates is essential for decision-making in Brazil, as problems related to the inferior quality of data on the cause of death were common issue in the 1990’s, which brought about interventions aimed at improving the quality of data^13^. The GBD (Global Burden of Disease) study is an international initiative that helps to improve epidemiological records and produce comparable estimates of many diseases and public health problems in different places and time periods^14^. 

The present study analyzed the GBD-2019 study’s estimates of suicide mortality in Brazil from 2000 to 2019. Aiming to understand the impact of demographic transition, the Brazilian adult (50+ years) mortality rates were compared with the global rates; with the rates of England, a high-income country with an older age structure; and with the rates of Mexico, a Latin-American middle-income country with a similar age-structure as Brazil. Looking for regional disparities, the suicide burden among older Brazilian adults (60+ years) within all of the Brazilian states was also compared during the same period. 

## METHODS

This is an ecological study that aimed to analyze the profile of suicide MR in Brazil between 2000 and 2019, based on the estimates from the GBD 2019 study, whose methodology has been thoroughly described and published^14^. The GBD-Brazil study is the result of the cooperation between the Institute of Health Metrics and Evaluation, the Brazilian Ministry of Health, and the Federal University of Minas Gerais. The GBD-Brazil network counts on more than 300 collaborators, providing data and critical evaluation of the estimates^15^ ^-^ ^16^.

Suicide was defined by codes X60-X64.9, X66-X83.9, and Y87.0 from the tenth edition of the ICD (International Classification of Diseases)^17^. The SIM (Mortality Information System, in English) and the IBGE (Brazilian Institute of Geography and Statistics, in English) provided the vital statistics^18^. In addition to the basic causes of death, suicide statistics consider immediate and intermediate causes in the death certificate^14^. Part of the garbage codes, X59 (exposure to non-specific factor) and Y34 (non-specific event, undetermined intention), were redistributed for suicide based on the description of the lesion in the death certificate^14^. Other codes, which are not considered the cause of death or are less specific - such as Y10-Y34 (external cause of undetermined intention) or R99 (other ill-defined causes and non-specific causes of mortality) - were redistributed based on the fraction of well-determined suicides in that category^14^.

The Brazilian national rates were compared to those from Mexico and England, two out of the 17 countries whose estimates were more comprehensively analyzed at the subnational level (Brazil, China, India, Indonesia, Italy, Japan, Kenya, Mexico, Nigeria, Pakistan, the Philippines, the Poland, Russia, South Africa, Sweden, the UK, and the US)^14^. Although developed countries have a vast literature on suicide, Mexico was the only Latin American country that published information of the burden of suicide^19^. The analysis between global rates and the comparator countries used age-standardized rates and two GBD groups of age-specific rates: 50-69 and 70+ age groups. 

The analysis for the Brazilian states considered the official definition of older adults in the country (60+ years)^20^. Estimates were evaluated by sex and age groups (60-64, 65-69, 70-74, and 80+ years). All locations were classified according to the SDI (Socio-Demographic Index), which ranges from 0 to 1 and is obtained from the weighted average of the *per capita* income, average educational attainment, and the fertility rate from each location^14^.

Mortality estimation used a large variety of possible models, including spatiotemporal Gaussian Process Regression models and mixed effects linear models to generate age-specific mortality estimates by all causes for the 204 countries^14^. The all-cause mortality data according to sex and age is organized before correcting for garbage codes and under-reporting of deaths, using a combination of life tables, death distribution methods, and regression techniques^14^. After obtaining the total number of deaths per year, the next step refers to the estimation of mortality by each cause of death, using the CODEm (Cause of Death Ensemble model), an analytical tool which associates several plausible models to produce a measurement with the best predictive value^14^. To obtain estimates for suicide, covariates were selected from medical literature and classified according to the level of association with suicide; those used the most for the model were *per capita* alcohol consumption, non-partner lifetime prevalence of sexual violence (female-only), prevalence of major depressive disorder, and population density^14^. 

Each estimate, according to location, sex, and age group, was calculated 1,000 times with data samples entered in the models and then distributed from the lowest to the highest value. The 95% uncertainty interval (UI) is obtained from the 2.5 and 97.5 percentiles of this distribution, and it considers the error generated by the sample, the modelling, and the availability of data^14^. All estimates are presented with a 95% UI, and the rates were described per 100,000 inhabitants. The differences between estimates were considered statistically significant if there was no coincidence of the 95% UI.

The time trend analysis between 2000 and 2019 estimated the number of deaths of individuals aged 60-79 years and 80+ years for each year, using the respective estimates of population size conducted by the GBD 2019^21^. After testing the overdispersion of the count data, the Poisson regression model was preferred over the model with Negative Binomial distribution and was used for sex and the general population. It was considered to be the offset to control the number of death cases within the population for each of the years^22^. The expected value was obtained by multiplying the rate observed for Brazil during the period from 2000 to 2019 by the population of each stratus (local and sex) evaluated in this study. The time trend (stable, increasing, or decreasing) was verified through the relative risk. The statistical significance considered the 95% confidence interval. To adjust the Poisson regression models, the RStudio software was used.

The estimates from this study are available at http://ghdx.healthdata.org/gbd-results-tool. The GBD study conforms to the Guidelines for Accurate and Transparent Health Estimates Reporting^23^. Secondary data was used, with no identification of individuals. Therefore, there was no need for informed consent. The GBD Brazil study was approved by the UFMG Research Ethics Committee (CAAE: 62803316.7.0000.5149) and is in accordance with the Declaration of Helsinki, reviewed in 2000. The authors have no conflicts to declare.

## RESULTS

In 2019, 13,500 (95% UI 12,800; 14,700) deaths by suicide were estimated in Brazil, and 759,000 (95% UI 685.400; 831.900) in the world. Of these deaths, 1,200 (95% UI 1,100; 1,300) and 113,700 (95% UI 100,200; 125,800) were of individuals over 70 years, which corresponds to 8.9% and 14.9% of the suicide deaths in these locations, respectively.

In 2000, 2010, and 2019, the global ASMR per 100,000 inhabitants were higher than in the three studied countries. In 2000, the global rates (14.43; 95% UI 13.02; 15.11) were twice as high as the Brazilian rates (6.53; 95% UI 6.27; 6.71). In 2000 and 2010, England had a higher ASMR, while Mexico presented a lower ASMR than Brazil. In 2019, Brazil and Mexico showed similar rates but slightly lower than the global rates and those from England (Table 1).

**TABLE 1:** Mortality rates by suicide worldwide, in Brazil, England, and Mexico, in 2000 and 2019. Estimates from Global Burden of Disease Study 2019.

2000			2010		2019		Δ
Age standardized							2010-2019
Locais	ASMR*	95% UI	ASMR	95% UI	ASMR	95% UI	(95% UI)
Global	14.43	13.02-15.11	11.24	10.21-12.05	9.39	8.48-10.29	-0.16 (-0.10. -0.21)
Brazil	6.53	6.27-6.71	6.04	5.88-6.18	5.68	5.40-6.19	-0.06 (0.03.-0.10)
Mexico	4.75	4.66-4.83	5.45	5.34-5.54	6.01	5.1-7.04	0.10 (0.28.-0.06)
England	8.21	8.03-8.35	6.89	6.78-7.04	6.63	6.43-6.95	-0.04 (0.00.-0.06)
**Age specific rates**							
**50-69 years-old**							
Global	22.15	19.95-23.39	17.61	15.92-18.67	14.25	12.76-15.60	-0.19 (-0.12-0.25)
Brazil	10.51	9.94-10.92	8.99	8.71-9.29	8.49	8.01-9.31	-0.06 (0.03.-0.11)
Mexico	5.74	5.6-5.89	6.16	6.01-6.32	6.51	5.31-7.95	0.06 (0.29.-0.13)
England	10.52	10.28-10.74	10.08	9.85-10.32	10.2	9.83-10.79	0.01 (0.06.-0.02)
**70+ years-old**							
Global	34.96	31.31-36.83	30.23	26.57-32.05	24.53	21.60-27.14	-0.19 (-0.13. -0.25)
Brazil	11.57	10.76-12.11	10.26	9.33-10.78	9.37	8.44-10.22	-0.09 (-0.01.-0.13)
Mexico	8.26	7.63-8.65	8.17	7.51-8.56	7.56	6.39-8.81	-0.07 (0.08.-0.20)
England	9.8	9.14-10.18	7.27	6.69-7.76	7.73	7.06-8.2	0.06 (0.09.0.03)

**ASMR:** Age standardized mortality rate; **UI:** Uncertainty interval.

The global mortality rates by age group showed an increasing gradient with aging, with less distinction between the studied countries. For instance, in 2019, the mortality rates per 100,000 inhabitants for those of 50-69 years were 1.5 higher in the world (14.25 95% UI 12.76; 15.60) than in Brazil (8.49, 95% UI 8.01; 9.31). Among the older adults (70+ years), this difference proved to be higher, 24.53 (95% UI 21.60; 27.14) globally, and 9.37 (95% UI 8.44; 10.22) in Brazil. England proved to be an exception, as it presented lower rates among the older adults: in 2019, the rates were 10.2 (95% UI 9.83; 10.79) for the 50-69 age group, and 7.73 (95% UI 7.06; 8.20) for the 70+ age group. Brazil was the country with the highest mortality rate for the 70+ age group in the three years studied. In 2019, the mortality rate for this age group was 9.37 (95% UI, 8.44; 10.22), as compared to the 7.56 (95% UI 6.39; 8.81) in Mexico and 7.73 (95% UI 7.06; 8.20) in England (Table 1).

In the 20 years studied, there was a decline in the ASMR globally. In the first 10 years of this period, this decline is also present in Brazil and in England, but not in Mexico. Meanwhile, between 2010 and 2019, a stability was observed in the ASMR of these countries, with a coincidence of a 95% UI for Brazil and Mexico, which presented ASMRs smaller than those found globally and in England. In this second decade, the decrease in the Brazilian rates was statistically significant only in the subgroup of 70+ years (-9%; 95% UI -1%; -13%), a reduction that was still lower than the global mortality rate (-19%; 95% UI -13%; -25%). In this age group, the Mexican rates showed no significant variation, whereas an increase of 6% (95% UI 3%; 9%) was verified in the English rate (Table 1).

**FIGURE 1:**
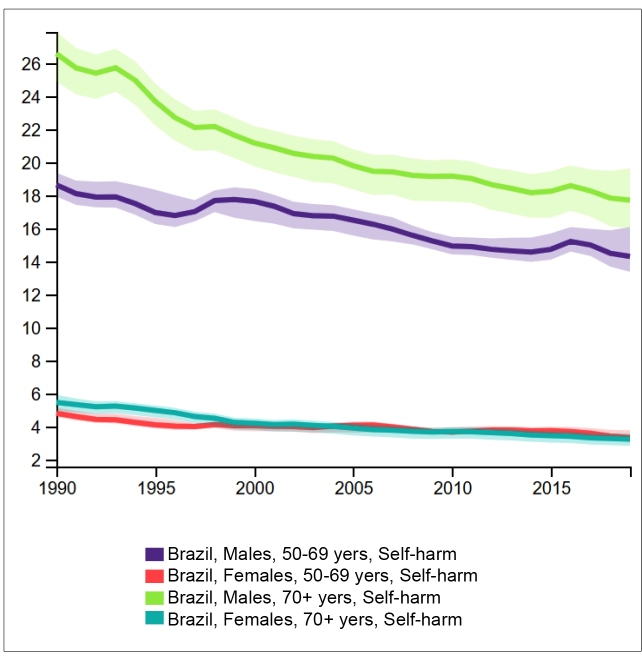
Annual estimates and uncertainty intervals of suicide deaths by 100,000 inhabitants in Brazil, by sex and age group, between 1990 and 2019. **Source:** IHME, GBD Study Results Tool: http://ghdx.healthdata.org/gbd-results-tool

In Brazil, the numbers of suicide deaths were higher for men than for women in all the age groups (Figure 1), with 981 (95% UI 886; 1087) male deaths and 246 (95% UI 215; 273) female deaths in the 70+ years age group in 2019, representing a sex ratio of 4:1.

Suplementary Material Tables1S **and** 2S show the mortality rates for the older adults by sex and age group, for Brazil and its states, in 2010 and 2019. A gradual aging increase was found in mortality for males but not for females. In 2019, among men aged 60 to 64 years, there were 13.88 (95% UI 12.67; 15.70) suicide deaths per 100,000 inhabitants, while for men with 80+ years this rate was equal to 20.25 (95% UI 17.09; 22.48). There was a significant variation in the mortality rates from different states. For example, in 2019, the mortality rates per 100,000 men, 60-64 years of age, varied from 7.24 (95% UI 5.31; 9.85) to 26.32 (95% UI 20.21; 34.50); three states showed mortality rates of below 10, with 22 states between 10 and 20, and only the states of Rio Grande do Sul and Santa Catarina with mortality rates above 20. Older men from Rio Grande do Sul (SDI=0.684) presented the highest suicide mortality rate in all of the age groups. Santa Catarina (SDI= 0.691) had the second highest rate in nearly all the age groups, in the two years studied. In 2019, two states with a below average SDI - Alagoas (SDI=0.518) and Pará (SDI=0.569) - were among the five lowest suicide rates for all age groups in the years studied **(** Suplementary Material Table 1S **)**.

Likewise it was observed for men, older women from Rio Grande do Sul had the highest estimates of suicide for all age groups, while Santa Catarina and Roraima (SDI=0.610) alternate between the second and third positions. Two states with a below average SDI in 2019 - Bahia (SDI=0.562) and Pará (SDI=0.569) - were consistently among the five states with the lowest rates of suicide for women **(** Suplementary Material Table 2S **)**.

Large 95% UI were observed for the states of the North and Northeast regions of Brazil, with statistically similar rates for most of these states. Alagoas and Pará had lower estimates, while Roraima had higher rates in comparison to the other states of their respective regions.

The time trend analysis of mortality among the older adults in Brazil revealed a statistically significant decline in the national rates between 2000 and 2019, with stability in the mortality rates in 15 states of Brazil. In 2019, there was a significant decrease in mortality in the states with higher SDIs: those from the Midwest and South regions, along with São Paulo and Rio de Janeiro, from the Southeast region. There was an increase in mortality rates in three states with an SDI of equal to or below the average in 2019 - Maranhão (SDI=0.444), Bahia (SDI=0.562), and Amapá (SDI=0.641) - the first two, only among men, and the third, among women (Table 2).

**TABLE 2:** Time trends in suicide mortality among older adults in Brazil, by sex and Federative Unit, between 2000 and 2019.

		Both sexes		Female			Male		
	RR*	95%CI	Trend	RR	95%CI	Trend	RR	95%CI	Trend
**Brazil**	0.989	0.987-0.991	Decreasing	0.989	0.985-0.992	Decreasing	0.990	0.988-0.992	Decreasing
**North Region**									
Acre	0.995	0.961-1.032	Stable	0.980	0.904-1.065	Stable	0.988	0.951-1.027	Stable
Amapá	1.000	0.957-1.046	Stable	1.160	1.007-1.399	Increasing	0.990	0.945-1.039	Stable
Amazonas	0.990	0.972-1.009	Stable	0.984	0.942-1.028	Stable	0.993	0.972-1.013	Stable
Pará	0.995	0.982-1.008	Stable	0.988	0.96-1.018	Stable	0.998	0.983-1.013	Stable
Rondônia	0.989	0.968-1.011	Stable	0.989	0.942-1.039	Stable	0.993	0.969-1.018	Stable
Roraima	0.991	0.949-1.036	Stable	1.045	0.941-1.179	Stable	0.990	0.944-1.040	Stable
Tocantins	1.013	0.991-1.035	Stable	0.991	0.942-1.044	Stable	1.017	0.993-1.042	Stable
**Northeast Region**									
Alagoas	1.003	0.984-1.022	Stable	1.002	0.965-1.041	Stable	1.003	0.981-1.026	Stable
Bahia	1.007	1.000-1.014	Increasing	0.995	0.977-1.013	Stable	1.011	1.004-1.019	Increasing
Ceará	1.001	0.993-1.008	Stable	1.000	0.984-1.017	Stable	1.002	0.993-1.010	Stable
Maranhão	1.016	1.004-1.027	Increasing	0.994	0.968-1.020	Stable	1.023	1.010-1.036	Increasing
Paraíba	1.002	0.989-1.015	Stable	0.995	0.969-1.022	Stable	1.006	0.991-1.021	Stable
Pernambuco	1.004	0.996-1.013	Stable	0.997	0.98-1.014	Stable	1.008	0.998-1.018	Stable
Piaui	0.992	0.979-1.004	Stable	0.991	0.964-1.019	Stable	0.992	0.979-1.006	Stable
Rio Grande do Norte	0.990	0.978-1.003	Stable	0.989	0.962-1.018	Stable	0.991	0.977-1.005	Stable
Sergipe	0.995	0.977-1.013	Stable	0.987	0.952-1.025	Stable	0.997	0.977-1.018	Stable
**Midwest Region**									
Distrito Federal	0.974	0.954-0.994	Decreasing	0.975	0.936-1.016	Stable	0.978	0.955-1.002	Stable
Goiás	0.983	0.974-0.992	Decreasing	0.981	0.961-1.002	Stable	0.985	0.975-0.995	Decreasing
Mato Grosso	0.981	0.965-0.997	Decreasing	0.983	0.947-1.020	Stable	0.982	0.964-1.000	Decreasing
Mato Grosso do Sul	0.979	0.966-0.993	Decreasing	0.978	0.949-1.009	Stable	0.982	0.967-0.997	Decreasing
**Southeast Region**									
Espírito Santo	0.994	0.979-1.009	Stable	0.986	0.957-1.017	Stable	0.996	0.979-1.013	Stable
Minas Gerais	0.999	0.994-1.004	Stable	0.998	0.987-1.010	Stable	0.999	0.993-1.005	Stable
Rio de Janeiro	0.980	0.974-0.986	Decreasing	0.986	0.974-0.999	Decreasing	0.977	0.971-0.984	Decreasing
São Paulo	0.977	0.974-0.981	Decreasing	0.980	0.972-0.988	Decreasing	0.977	0.973-0.981	Decreasing
**South Region**									
Paraná	0.985	0.979-0.992	Decreasing	0.983	0.968-0.999	Decreasing	0.988	0.98-0.995	Decreasing
Rio Grande do Sul	0.985	0.981-0.989	Decreasing	0.988	0.979-0.999	Decreasing	0.983	0.978-0.988	Decreasing
Santa Catarina	0.980	0.972-0.987	Decreasing	0.985	0.969-1.002	Stable	0.977	0.969-0.985	Decreasing

*RR: Relative risk, **CI:** confidence interval.

## DISCUSSION

The present study revealed that Brazil presented a lesser decline in ASMRs than worldwide, with a male predominance among men and rates increasing with aging, between 2000 and 2019. Most of the states with SDI above the national average presented higher rates throughout the period, and two states with lower SDIs presented an increase in mortality rates of older men.

The estimates found are similar to previous studies, which also showed lower ASMRs in Latin American countries when compared to global suicide mortality rates, with a predominance in males and an increase with age^24^. Other studies in Brazil also showed that suicide mortality rates were lower than global mortality rates^6^ ^,^ ^25^ ^-^ ^29^, in addition to the large variation among states^25^ ^,^ ^30^ ^,^ ^31^. Differences in the magnitude of the results can be attributed to methodological heterogeneity, but the direction of the results is the same. 

In Brazil, the demographic transition seems not to explain the higher rates among individuals over 70 years of age, since these rates were higher when compared to England and Mexico, countries with an older and younger age structure, respectively. Demographic transition is a recent phenomenon in Brazil, which still has a predominantly young population^11^. Therefore, specific interventions for older adults are necessary to prevent an increase in these rates with population aging. Health policies for the older adults in Brazil are not still consolidated or have had little time to produce a true impact. In England, a reduction of suicide mortality was already taking place, especially among men over 55 years of age, associated with an increase in the rates among younger men^33^. This evolution is related to an improvement in health care for the older adults, as well as an increase in the unemployment and divorce rates, an increase in the consumption of alcohol and other drugs, and a reduction in the number of marriages in the country^33^. In Mexico, the economic crisis is responsible for the higher suicide rates among the young than among older adults^19^. It is worth mentioning that the pace of the reduction of suicide rates in the three studied countries, between 2010 and 2019, has been below that needed to reach the objective of a 30% reduction by 2030^2^, and if this pace does not change, the goal will not be reached. 

The suicide mortality rates found in this study are higher than those verified in the country to date^6^ ^,^ ^25^ ^-^ ^29^, most likely because no previous study has made any correction for the under-reporting of deaths, and only two have used any method of correction for garbage codes^32^ ^,^ ^34^. The decline in these rates in the period studied is compatible with the tendency already demonstrated by the GBD 2015^35^, but it is contradictory to 11 studies which identified an increase in mortality in recent decades^6^ ^,^ ^25^ ^,^ ^26^ ^,^ ^28^ ^-^ ^32^ ^,^ ^34^ ^,^ ^36^ ^,^ ^37^. Seven studies failed to show the proper ASMRs; therefore, the increase in these rates over time might be due only to the process of aging within the Brazilian population^6^ ^,^ ^25^ ^,^ ^28^ ^,^ ^30^ ^,^ ^31^ ^,^ ^34^ ^,^ ^37^. Of the four studies that used ASMRs^26^ ^,^ ^29^ ^,^ ^31^ ^,^ ^36^, only one implemented a correction for mortality garbage codes^31^ and two refer to the periods of 1980 to 2005 and 1990 to 2015^26^ ^,^ ^31^.

Only six Brazilian studies were found that focus on older adults’ suicide in the last two decades^5^ ^-^ ^10^, three of which were subnational (one in Minas Gerais^5^ and two in Bahia^8^ ^,^ ^9^), and only one of which covered a period similarly to that of the present study^10^. Unlike the GBD 2019 estimates, these studies identified an increase in the national or subnational suicide mortality rates among the older adults. Again, the methodological differences may well explain these differences: none of these studies corrected for under-registration or redistributed garbage-codes^5^ ^-^ ^10^, and three used ASMR^7^ ^,^ ^8^ ^,^ ^10^. In accordance with this study, higher rates were identified in the South region^7^ ^,^ ^10^, as compared to lower rates in the North region of the country^10^ and in the state of Rio de Janeiro^6^ ^,^ ^7^. 

The subnational analysis revealed that most states with higher social economic development, with SDIs above the national average, showed the highest mortality rates, different from other studies that show a connection between a worse socioeconomic status and a higher mortality due to this cause^38^. This result is consistent with the fact that the states with the lowest SDI are also those with a poorer quality of information^39^. Although Brazil is classified as four out of five stars in terms of mortality data completion, the death certificates still show high percentages of garbage codes, with a reduction from 25% to 17% during the period of this study^14^. Suicides are commonly classified as deaths with undetermined intention (codes CID-10 Y10-Y34), accidents (codes V01-X59), homicides (codes X85-Y09), and unknown cause (codes R95-R99)^1^. These garbage codes still compromise the quality of the estimates in Brazil, especially for the older adults^39^ ^,^ ^40^ and in the North and Northeast regions of the country^39^. The poor quality of records is more frequent in small towns, which usually do not have a Coroner’s Office^41^. Initiatives seeking to integrate the data from SIM with information from other departments, such as the SAMU (Mobile Emergency Care Service, in English), Coroner’s Offices, and the written press have been useful to increase the quality of death statistics^39^ ^,^ ^42^ ^,^ ^43^.

High rates of suicide in the South region of Brazil have been identified in other studies^44^ ^,^ ^45^. The state of Rio Grande do Sul showed the highest rates in the country between 1980 and 1999^44^. The use of pesticides and the tobacco industry have been suggested as a main cause, but with no confirmation^45^ ^-^ ^47^. Other previously described risk factors include widowhood, work as fishermen^44^, in addition to lower income, less schooling, and less urbanization^45^.

The ratio of nearly four times as many deaths among older men as among older women in Brazil is close to the pattern in high-income countries^1^. Among the factors suggested to explain this are the higher frequency of violent attempts and higher frequency of disorders caused by alcohol consumption among men, besides the fact that women are more likely to search for psychiatric treatment^1^.

Determining risk factors and the causality chain of suicide is beyond the limits of this ecological study. Suicide among older adults has a broad net of health determinants that may be related to the society (access to education^45^ ^,^ ^49^ ^,^ ^50^, poverty^38^, access to lethal means^1^, inappropriate media reporting^1^ ^,^ ^48^), to the community (social isolation^48^ ^,^ ^51^, lack of social support^49^ ^,^ ^51^ ^-^ ^53^, grieving^51^, exposure to violence, discrimination, trauma, and abuse^48^), as well as to barriers to adequate healthcare^1^. 

This study has, as a strength, a standardized methodology, which allowed for the detection of regional variations and variations over time, the correction of the under-reporting of deaths, and redistribution of garbage codes. Despite that, statistical correction methods are not perfect, and vital data must have its quality improved to better characterize the frequency of suicides. The evaluation of the temporal tendency among the states of Brazil did not consider the uncertainty intervals of the estimates, which might compromise the interpretation of the results. Moreover, this study did not evaluate factors other than sex and age, which had already been identified in Brazil, such as having indigenous ethnicity^48^ ^,^ ^54^ and having low level education^50^, which certainly influenced the heterogeneity of results. The only suicide method that the GBD 2019 allows to evaluate separately is the use of firearms, which limits the planning of preventive strategies focused on this aspect. Unfortunately, the GBD age groups (50-69 and 70+ years) are not coincident with the usual definition of middle-aged and older adults^20^ ^,^ ^55^, preventing further comparability.

Every approach in terms of health must be based on evidence. However, the scientific studies on suicide prevention are few, which shows the difficulty in studying the problem. As it is not a frequent event, the number of participants in clinical studies and of cohorts must be high in order to make the results statistically significant^56^. Moreover, since it is multifactorial and strongly influenced by cultural aspects, the approaches are usually heterogeneous and multimodal, making it difficult to specify which component of the prevention program is more efficient^56^ ^-^ ^57^.

In conclusion, national prevention strategies must monitor not only the deaths, but also the suicide attempts, as well as identify vulnerable groups, promote protection factors, improve access to psychiatric treatment, make the population aware of the problem, invest in public education to eliminate the stigma about mental disorders and against individuals with suicidal behaviour, and encourage the media to adopt better practices in relation to the reports of suicide^56^ ^-^ ^57^.
