# Supplementary material for: Suicide mortality among older adults in Brazil between 2000 and 2019 - estimates from the Global Burden of Disease Study 2019
Source: Rev Soc Bras Med Trop. 2022 Jan 28;55(Suppl 1):e0322-2021. doi: 10.1590/0037-8682-0322-2021 (PMC9009432; doi:10.1590/0037-8682-0322-2021)
Supplement: Supplementary file 1 [file 1678-9849-rsbmt-55-s01-e0322-2021-supp1.pdf]

TABLE 1S: Mortality rates by suicide among older men in Brazil and Federative Units, in 2010 and 2019. Estimates from the Global Burden of Disease Study 2019.

	Age-specific mortality rate by 100,000 inhabitants (95%UI)									
	2010					2019				
**Age (years)**	60-64	65-69	70-74	75-79	80+	60-64	65-69	70-74	75-79	80+
**Local (2019 SDI)**										
**Brazil** SDI: 0.64	14.66 (13.88-15.42)	15.5 (14.64-16.36)	17.13 (16.03-18.12)	18.79 (17.45-20.04)	22.55 (19.52-24.41)	13.88 (12.67-15.70)	14.87 (13.63-16.73)	16.15 (14.75-18.19)	17.45 (15.81-19.41)	20.25 (17.09-22.48)
**North Region**										
Acre SDI: 0.562	13.16 (10.51-16.8)	14.01 (10.74-17.93)	16.71 (13.08-21.44)	21.54 (16.28-27.45)	29.2 (24.01-34.77)	13.19 (9.74-17.6)	13.54 (9.89-18.16)	17.77 (13.49-23.32)	20.74 (15.64-27.07)	29.81 (24.01-37)
Amapá SDI: 0.641	10.6 (8.24-13.76)	13.61 (10.50-17.45)	16.15 (12.71-20.58)	18.21 (13.99-23.39)	21.89 (17.55-26.34)	12.74 (9.26-16.69)	13.81 (10.3-18.40)	17.31 (13.05-22.58)	17.65 (12.94-22.80)	23.57 (18.72-28.77)
Amazonas SDI: 0.602	10.98 (8.52-13.97)	10.77 (8.34-13.62)	13.11 (10.19-16.54)	14.19 (10.89-17.84)	17.47 (13.75-21.20)	10.85 (8.13-14.24)	11.2 (8.28-14.61)	11.64 (8.80-15.21)	14.05 (10.52-18.16)	16.43 (12.62-20.06)
Pará SDI: 0.569	8.92 (6.63-11.72)	9.16 (6.77-12.06)	10.8 (7.33-13.48)	11.86 (8.72-15.82)	12.04 (9.23-14.73)	8.67 (6.25-11.63)	9.94 (7.24-13.36)	10.53 (7.59-14.26)	11.57 (8.22-15.57)	11.87 (9.11-14.92)
Rondônia SDI: 0.606	11.1 (8.65-14.26)	12.68 (9.82-16.28)	16.46 (12.64-20.86)	18.41 (14.10-23.57)	19.99 (15.70-24.71)	12.2 (8.84-16.65)	14.23 (10.09-19.01)	16.41 (11.84-22.43)	17.77 (13.06-23.68)	21.43 (16.69-26.60)
Roraima SDI: 0.610	14.5 (11.35-18.33)	17.31 (13.49-21.49)	19.68 (15.20-24.77)	23.81 (18.21-31.23)	36.28 (29.53-43.60)	16.6 (12.31-21.79)	18.82 (14.03-24.44)	20.25 (15.17-26.39)	21.84 (16.37-29.38)	34.23 (27.49-42.05)
Tocantins SDI: 0.583	12.98 (9.87-16.81)	15.22 (11.46-19.50)	17.12 (12.85-22.10)	18.01 (13.52-23.49)	23.15 (18.40-27.79)	15.61 (10.89-21.63)	17.29 (12.38-23.14)	20.5 (14.89-27.67)	21.18 (15.68-28.20)	31.56 (25.01-39.39)

**Northeast Region**										
Alagoas SDI: 0.518	9.05 (7.03-11.5)	8.66 (6.8-10.87)	9.42 (7.29-12)	10.28 (7.85-13.2)	10.79 (8.55-13)	8.86 (6.51-11.83)	8.51 (6.09-11.43)	9.81 (7.21-13.17)	10.73 (7.76-14.33)	11.74 (8.91-14.44)
Bahia SDI: 0.562	14.69 (11.45-18.42)	14.91 (11.67-18.92)	16.71 (12.91-21.46)	18.36 (14.09-23.77)	17.78 (14.05-21.40)	15.3 (10.87-21.51)	16.74 (11.57-23.10)	17.71 (12.28-24.34)	18.25 (13.15-24.89)	16.91 (13.09-21.15)
Ceará SDI: 0.558	16.94 (12.96-21.53)	16.64 (12.52-21.46)	17.75 (13.24-22.78)	21.25 (15.88-27.81)	24.74 (19.76-29.80)	18.43 (12.36-25.75)	18.58 (12.69-26.16)	20.07 (13.68-27.83)	21.55 (14.77-29.90)	25.91 (19.68-33.07)
Maranhão SDI: 0.444	10.79 (7.78-14.71)	10.64 (7.62-14.78)	12.31 (8.94-16.50)	12.22 (8.59-16.34)	15.23 (11.87-18.87)	13.83 (9.42-19.36)	15.75 (11.12-21.70)	16.33 (11.13-22.61)	16.27 (11.48-22.40)	22.22 (17.47-27.13)
Paraíba SDI: 0.548	13.54 (10.63-16.92)	13.54 (10.67-16.82)	13.72 (10.91-17.35)	14.96 (11.50-19.26)	17.22 (13.81-20.50)	13.3 (9.68-17.81)	13.41 (9.73-18.00)	13.79 (10.01-18.65)	14.54 (10.55-19.55)	15.54 (11.79-19.24)
Pernambuco SDI: 0.571	12.78 (10.46-15.37)	13.34 (11.10-16.05)	15.53 (12.67-18.84)	15.54 (12.58-19.43)	18.3 (14.99-21.60)	14.73 (10.92-19.09)	15.86 (12.14-20.45)	17.72 (13.36-22.89)	17.73 (13.24-22.76)	19.41 (15.61-24.07)
Piauí SDI: 0.509	14.67 (11.62-18.34)	17.09 (13.48-21.16)	17.6 (13.72-22.29)	20.06 (15.17-25.49)	25.93 (20.73-30.93)	16.01 (11.65-21.86)	16.43 (11.72-22.36)	18.32 (12.81-24.55)	19.02 (13.58-25.58)	22.76 (17.24-28.67)
Rio Grande do Norte SDI: 0.576	15.97 (11.92-21.18)	16.28 (11.95-21.39)	18.12 (13.23-23.91)	17.98 (13.21-23.71)	24.31 (18.95-29.59)	17.25 (12.12-23.99)	16.9 (11.67-23.70)	18.22 (12.50-25.11)	18.19 (12.33-24.81)	22.64 (16.66-29.20)
Sergipe SDI: 0.583	13.83 (10.57-18.07)	15.67 (12.11-19.84)	16.86 (12.83-21.79)	17.1 (12.93-22.33)	21.33 (16.71-25.54)	13.99 (10.10-19.04)	16.14 (11.45-21.77)	16.8 (12.08-22.81)	16.28 (11.79-21.83)	20.81 (15.78-25.79)
**Midwest Region**										
Distrito Federal SDI: 0.777	9.63 (7.37-12.10)	10.77 (8.46-13.48)	13.83 (10.89-17.34)	16.24 (12.51-20.67)	30.5 (25.58-36.32)	7.24 (5.31-9.85)	9.05 (6.65-12.05)	10.65 (7.84-14.42)	13.04 (9.63-17.62)	22.85 (18.03-28.60)
Goiás SDI: 0.628	17.83 (14.23-22.26)	18.3 (14.61-22.90)	20.85 (16.49-26.23)	23.36 (17.97-29.80)	28.23 (22.63-34.37)	15.68 (11.42-21.51)	17.45 (12.46-23.57)	20.04 (14.25-26.88)	23.99 (17.59-32.02)	25.4 (19.14-32.23)

Mato Grosso SDI 0.642	13.11 (10.58-16.09)	13.44 (10.79-16.75)	15.72 (12.51-19.87)	16.98 (13.50-21.42)	18.24 (14.79-21.81)	10.16 (7.48-13.50)	11.52 (8.50-15.38)	12.91 (9.51-16.96)	14.74 (11.00-19.79)	15.75 (12.27-19.82)
Mato Grosso do Sul SDI: 0.639	16.72 (13.42-20.75)	19.77 (15.95-24.62)	22.26 (17.81-27.66)	24.37 (19.02-30.32)	30.42 (25.18-36.22)	15.82 (11.18-21.47)	16.93 (12.80-22.69)	18.1 (13.51-23.46)	20.74 (15.33-27.91)	25.3 (19.72-31.77)
**Southeast Region**										
Espírito Santo SDI: 0.660	11.66 (9.35-14.57)	10.96 (8.71-13.66)	12.43 (9.85-15.83)	13.22 (10.28-17.00)	16.01 (12.96-19.11)	11.06 (8.17-14.69)	10.45 (7.75-13.92)	12.06 (8.87-16.27)	12.52 (9.32-16.51)	15.13 (11.80-18.81)
Minas Gerais SDI: 0.643	15.25 (13.04-17.79)	15.8 (13.23-18.51)	17.1 (14.27-20.13)	15.85 (13.08-18.89)	19.66 (16.13-22.70)	15.21 (11.66-19.25)	15.51 (12.11-19.74)	14.65 (11.25-18.48)	15.16 (11.57-19.34)	16.68 (13.15-20.08)
Rio de Janeiro SDI: 0.702	10.19 (8.58-12.00)	11.18 (9.41-13.22)	13.2 (10.72-15.76)	17.2 (14.42-20.56)	20.31 (17.15-23.34)	9.58 (7.31-12.41)	10.15 (7.67-12.78)	11.81 (9.04-15.55)	14.28 (10.92-18.37)	17.22 (13.75-21.15)
São Paulo SDI: 0.702	12.25 (10.62-14.01)	12.38 (10.64-14.21)	12.4 (10.45-14.66)	14.51 (12.21-16.90)	20.5 (17.24-23.40)	9.93 (7.64-12.79)	10.15 (7.99-13.02)	10.61 (8.21-13.65)	11.63 (8.83-14.93)	16.6 (13.33-19.81)
**South Region**										
Paraná SDI: 0.662	14.14 (11.88-16.65)	16.68 (13.98-19.56)	17.22 (14.43-20.75)	19.12 (15.78-23.05)	25.15 (20.86-29.24)	14.49 (10.96-18.66)	16.22 (12.55-20.79)	17.88 (13.64-23.05)	18.3 (13.97-23.60)	21.51 (17.16-26.09)
Rio Grande do Sul SDI: 0.684	29.02 (25.06-33.55)	33.72 (28.89-38.85)	40.49 (34.83-46.64)	43.19 (37.07-50.51)	48.38 (40.73-55.60)	26.32 (20.21-34.50)	29.68 (22.88-38.21)	34.51 (26.86-45.40)	39.33 (30.36-50.90)	39.99 (31.87-49.25)
Santa Catarina SDI: 0.691	24.96 (20.85-29.26)	25.41 (21.26-29.93)	30.31 (25.19-36.46)	33.97 (27.97-40.90)	40.81 (33.94-47.72)	21.74 (16.83-28.38)	24.29 (18.55-31.34)	26.86 (20.20-34.93)	28.02 (21.59-36.15)	30.99 (24.23-38.97)

SDI: Social demographic index
Colors: TxM < 10.00; TxM: 10-14.99; TxM: 15.00-19.99; TxM ≥ 20.00.
