# Supplementary material for: Suicide mortality among older adults in Brazil between 2000 and 2019 - estimates from the Global Burden of Disease Study 2019
Source: Rev Soc Bras Med Trop. 2022 Jan 28;55(Suppl 1):e0322-2021. doi: 10.1590/0037-8682-0322-2021 (PMC9009432; doi:10.1590/0037-8682-0322-2021)
Supplement: Supplementary file 2 [file 1678-9849-rsbmt-55-s01-e0322-2021-supp2.pdf]

TABLE 2S: Mortality rates by suicide among older women in Brazil and Federative Units, in 2010 and 2019. Estimates from the Global Burden of Disease Study 2019.

	Age specific mortality rate by 100,000 inhabitants (95% UI)									
			2010					2019		
**Age (years)**	60-64	65-69	70-74	75-79	80+	60-64	65-69	70-74	75-79	80+
**Locals (2019 SDI)**										
**Brazil** SDI: 0.64	3.29 (3.63-3.03)	3.46 (3.17-3.76)	3.53 (3.20-3.86)	3.56 (3.17-3.88)	4.05 (3.37-4.48)	3.07 (2.76-3.54)	3.16 (2.85-3.58)	3.12 (2.79-3.53)	3.07 (2.71-3.46)	3.54 (2.88-3.95)
**North Region**										
Acre SDI: 0.562	2.93 (2.13-3.91)	2.84 (2.11-3.78)	2.83 (2.11-3.72)	2.8 (2.09-3.73)	3.17 (2.48-3.87)	2.65 (1.89-3.59)	3.02 (2.23-4.02)	3.06 (2.17-4.11)	3.09 (2.19-4.12)	3.44 (2.63-4.24)
Amapá SDI: 0.641	2.43 (1.81–1.85)	2.57 (1.85–3.38)	3.1 (2.32–4.14)	2.62 (1.93–3.49)	3.1 (2.33–3.83)	2.83 (2.04–3.80)	3.17 (2.33-4.24)	3.02 (2.17-4.04)	2.97 (2.13-3.95)	3.32 (2.54-4.02)
Amazonas SDI: 0.602	2.31 (1.72-3.07)	2.5 (1.86-3.28)	3 (2.25-3.97)	2.85 (2.11-3.72)	3.08 (2.36-3.76)	2.15 (1.55-2.93)	2.31 (1.64-3.09)	2.63 (1.82-3.68)	2.77 (1.96-3.80)	2.87 (2.17-3.58)
Pará SDI: 0.569	1.93 (1.40-2.64)	2.15 (1.51-2.96)	2.37 (1.78-3.18)	2.43 (1.82-3.18)	2.59 (1.95-3.23)	1.93 (1.35-2.70)	2.07 (1.48-2.81)	2.19 (1.60-2.91)	2.36 (1.72-3.22)	2.45 (1.83-3.04)
Rondônia SDI: 0.606	3.69 (2.75-4.97)	4.29 (3.19-5.57)	4.66 (3.39-6.15)	4.99 (3.72-6.40)	4.88 (3.80-5.95)	3.53 (2.42-4.89)	3.94 (2.71-5.46)	4.45 (3.05-6.10)	4.8 (3.42-6.54)	4.84 (3.70-5.99)
Roraima SDI: 0.610	4.26 (3.15-5.74)	4.81 (3.52-6.24)	4.55 (3.33-6.13)	4.6 (3.36-6.10)	6.12 (4.90-7.54)	4.43 (6.08-3.19)	4.63 (3.33-6.39)	4.83 (3.49-6.63)	4.93 (3.51-6.74)	6.15 (4.74-7.59)
Tocantins SDI: 0.583	2.88 (2.08-3.80)	3.15 (2.26-4.19)	3.68 (2.68-5.00)	3.63 (2.69-4.76)	3.5 (2.68-4.34)	3.11 (2.18-4.32)	3.43 (2.35-4.75)	3.66 (2.62-5.00)	3.58 (2.54-4.99)	3.69 (2.77-4.64)

**Northeast Region**										
Alagoas<br>SDI: 0.518	2.46<br>(1.81-3.31)	2.57<br>(1.89-3.48)	2.65<br>(1.99-3.60)	2.58<br>(1.94-3.44)	2.55<br>(1.98-3.10)	2.48<br>(1.74-3.50)	2.63<br>(1.85-3.62)	2.69<br>(1.85-3.66)	2.74<br>(1.97-3.74)	2.72<br>(2.08-3.46)
Bahia<br>SDI: 0.562	2.12<br>(1.58-2.86)	2.11<br>(1.58-2.83)	2.03<br>(1.54-2.64)	1.95<br>(1.43-2.57)	2.12<br>(1.54-2.64)	2.1<br>(1.45-2.86)	2.11<br>(1.41-2.99)	1.95<br>(1.40-2.63)	1.86<br>(1.30-2.56)	2.17<br>(1.59-2.74)
Ceará<br>SDI: 0.558	3.71<br>(2.71-5.00)	3.78<br>(2.82-5.00)	3.86<br>(2.89-5.00)	3.86<br>(2.88-5.06)	4.5<br>(3.39-5.55)	3.61<br>(2.45-5.15)	4.33<br>(3.02-6.07)	3.73<br>(2.65-5.19)	3.76<br>(2.67-5.15)	4.32<br>(3.22-5.54)
Maranhão<br>SDI: 0.444	2.49<br>(1.79-3.45)	2.26<br>(1.63-3.15)	2.51<br>(1.84-3.41)	2.33<br>(1.70-3.12)	2.65<br>(1.98-3.29)	2.6<br>(1.68-3.90)	2.28<br>(1.55-3.25)	2.52<br>(1.79-3.45)	2.38<br>(1.75-3.20)	2.77<br>(2.10-3.52)
Paraíba<br>SDI: 0.548	2.75<br>(2.10-3.60)	3.06<br>(2.31-4.02)	3.05<br>(2.25-3.99)	3.17<br>(2.33-4.16)	3.49<br>(2.67-4.19)	2.73<br>(1.91-3.78)	3.06<br>(2.23-4.06)	2.95<br>(2.13-4.02)	2.94<br>(2.12-4.01)	3.43<br>(2.64-4.27)
Pernambuco<br>SDI: 0.571	3.16<br>(2.47-4.08)	3.26<br>(2.47-4.25)	3.41<br>(2.59-4.43)	3.48<br>(2.62-4.60)	4.04<br>(3.20-4.89)	3.12<br>(2.20-4.17)	3.6<br>(2.60-4.83)	3.58<br>(2.60-4.84)	3.65<br>(2.66-4.84)	3.87<br>(3.04-4.87)
Piauí<br>SDI: 0.509	3.44<br>(2.57-4.51)	3.4<br>(2.53-4.39)	3.88<br>(2.89-4.99)	3.91<br>(2.96-5.04)	4.28<br>(3.18-5.20)	3.51<br>(2.42-4.93)	3.78<br>(2.60-5.27)	3.75<br>(2.71-5.05)	3.8<br>(2.74-5.04)	3.95<br>(2.83-4.95)
Rio Grande do Norte<br>SDI: 0.576	3.04<br>(2.20-4.10)	3.45<br>(2.52-4.60)	3.44<br>(2.58-4.45)	3.73<br>(2.78-4.89)	4.09<br>(3.05-5.05)	3.06<br>(2.10-4.26)	3.56<br>(2.49-4.98)	3.4<br>(2.43-4.55)	3.61<br>(2.56-4.83)	3.93<br>(2.86-4.96)
Sergipe<br>SDI: 0.583	3.33<br>(2.47-4.48)	3.87<br>(2.89-5.13)	3.8<br>(2.69-5.13)	3.97<br>(2.95-5.26)	4.16<br>(3.15-5.07)	3.3<br>(2.23-4.61)	3.61<br>(2.54-5.05)	3.95<br>(2.70-5.50)	3.84<br>(2.59-5.26)	4.19<br>(3.21-5.31)
**Midwest Region**										
Distrito Federal<br>SDI: 0.777	2.37<br>(1.79-3.14)	3.03<br>(2.24-3.99)	3.31<br>(2.49-4.34)	3.76<br>(2.74-4.91)	5.32<br>(4.29–6.36)	2.22<br>(1.57-3.05)	2.48<br>(1.77-3.40)	2.94<br>(2.10-4.04)	3.06<br>(2.19-4.16)	4.48<br>(3.48–5.57)

Goiás SDI: 0.628	3.55 (2.69-4.65)	3.97 (2.95-5.13)	4.35 (3.13-5.81)	4.63 (3.45–6.18)	5.68 (4.41–6.98)	3.86 (2.70-5.36)	3.86 (2.72-5.31)	3.78 (2.54-5.29)	4.43 (2.99–6.09)	5 (3.81–6.30)
Mato Grosso SDI 0.642	2.91 (2.14-3.80)	3.58 (2.68-4.59)	3.79 (2.82-5.00)	4.1 (3.06-5.31)	4.27 (3.40-5.18)	2.79 (2.00-3.84)	3.13 (2.22-4.31)	3.38 (2.42-4.61)	3.38 (2.41-4.57)	3.66 (2.82-4.51)
Mato Grosso do Sul SDI: 0.639	4.46 (3.37-5.94)	4.72 (3.45-6.04)	4.81 (3.54-6.14)	5.17 (3.90-6.81)	6.11 (4.78-7.31)	3.76 (2.70-5.15)	3.85 (2.74-5.27)	4.12 (2.84-5.68)	4.38 (3.04-5.96)	5.03 (3.80-6.32)
**Southeast Region**										
Espírito Santo SDI: 0.660	2.5 (1.88-3.25)	2.66 (1.99-3.50)	2.95 (2.26-3.94)	2.94 (2.18-3.85)	3.64 (2.86-4.34)	2.57 (1.80-3.46)	2.69 (1.88-3.60)	2.81 (1.98-3.84)	2.82 (2.02-3.72)	3.27 (2.51-4.03)
Minas Gerais SDI: 0.643	3.86 (3.11-4.74)	3.89 (3.16-4.78)	3.42 (2.67-4.34)	3.52 (2.75-4.47)	3.83 (3.07-4.55)	3.63 (2.66-4.76)	3.59 (2.66-4.74)	3.08 (2.30-4.14)	2.74 (2.02-3.65)	3.14 (2.37-3.83)
Rio de Janeiro SDI: 0.702	2.51 (1.98-3.17)	2.79 (2.20-3.46)	2.95 (2.27-3.78)	3.17 (2.40-4.04)	3.29 (2.60-3.97)	2.36 (1.76-3.10)	2.42 (1.80-3.25)	2.46 (1.79-3.32)	2.67 (1.99-3.46)	2.87 (2.24-3.50)
São Paulo SDI: 0.702	3.07 (2.55-3.68)	3.03 (2.48-3.66)	3.2 (2.60-3.92)	3.24 (2.57-3.97)	4 (3.22-4.75)	2.4 (1.78-3.22)	2.35 (1.76-3.08)	2.27 (1.69-3.10)	2.24 (1.67-2.98)	2.97 (2.34-3.60)
**South Region**										
Paraná SDI: 0.662	3.15 (2.47-3.99)	3.89 (3.02-4.90)	3.61 (2.77-4.63)	3.34 (2.56-4.30)	4.49 (3.67-5.41)	3 (2.16-4.10)	3.29 (2.40-4.35)	3.47 (2.50-4.61)	2.93 (2.15-3.96)	3.94 (3.12-4.83)
Rio Grande do Sul SDI: 0.684	6.05 (4.80-7.37)	6.36 (5.09-7.84)	6.58 (5.25-8.08)	6.47 (5.11-8.17)	7.34 (5.81-8.63)	5.95 (4.32-7.93)	5.93 (4.40-7.77)	6.19 (4.59-8.34)	6.13 (4.48-8.02)	6.35 (4.85-7.82)
Santa Catarina SDI: 0.691	4.84 (3.73-6.16)	5.34 (4.07-6.72)	5.73 (4.43-7.25)	5.01 (3.80-6.42)	6.5 (5.18-7.72)	4.96 (3.63-6.45)	5.05 (3.67-6.77)	4.77 (3.48-6.53)	4.64 (6.35-3.36)	5.89 (4.63-7.22)

***SDI:** Social demographic index
Color legend: TxM <5 TxM: 5.00-8.00
